# Kinetic model of metabolic network for xiamenmycin biosynthetic optimisation

**DOI:** 10.1049/iet-syb.2014.0054

**Published:** 2016-02-01

**Authors:** Min‐juan Xu, Yong‐cong Chen, Jun Xu, Ping Ao, Xiao‐mei Zhu

**Affiliations:** ^1^ Shanghai Center for Systems Biomedicine, Key Laboratory of Systems Biomedicine Shanghai Jiao Tong University Shanghai 200240 People's Republic of China; ^2^ SmartWin Technology 67 Tranmere Ave, Carnegie VIC Melbourne 3163 Australia; ^3^ Institute of Oceanology, Shanghai Jiao Tong University Shanghai 200240 People's Republic of China; ^4^ State Key Laboratory of Microbial Metabolism and School of Life Sciences and Biotechnology, Shanghai Jiao Tong University Shanghai 200240 People's Republic of China; ^5^ GeneMath 5525 27th Ave. N.E. Seattle WA 98105 USA

**Keywords:** enzymes, biochemistry, microorganisms, pharmaceuticals, genetics, Lyapunov methods, optimisation, kinetic model, metabolic network, xiamenmycin biosynthetic optimisation, prenylated benzopyran compounds, antifibrotic bioactivity, mangrove‐derived Streptomyces xiamenensis, kinetic metabolic model, Streptomyces lividans, xiamenmycin‐oriented genetic modification, generic enzymatic rate equations, Lyapunov function, kinetic model, flux distributions, S. lividans, glucose, glycerol, carbon sources, bacterium, xiamenmycin production, phosphoenolpyruvate synthesis, citric acid cycle

## Abstract

Xiamenmycins, a series of prenylated benzopyran compounds with anti‐fibrotic bioactivities, were isolated from a mangrove‐derived *Streptomyces xiamenensis*. To fulfil the requirements of pharmaceutical investigations, a high production of xiamenmycin is needed. In this study,, the authors present a kinetic metabolic model to evaluate fluxes in an engineered *Streptomyces lividans* with xiamenmycin‐oriented genetic modification based on generic enzymatic rate equations and stability constraints. Lyapunov function was used for a viability optimisation. From their kinetic model, the flux distributions for the engineered *S. lividans* fed on glucose and glycerol as carbon sources were calculated. They found that if the bacterium can utilise glucose simultaneously with glycerol, xiamenmycin production can be enhanced by 40% theoretically, while maintaining the same growth rate. **G**lycerol may increase the flux for phosphoenolpyruvate synthesis without interfering citric acid cycle. They therefore believe this study demonstrates a possible new direction for bioengineering of *S. lividans*.

## 1 Introduction

Xiamenmycins, a series of prenylated benzopyran compounds, were isolated from a mangrove‐derived *Streptomyces xiamenensis* [1–3]*. Streptomyces* are well‐known microbial cell factory for bioactive secondary metabolites based on various biosynthetic pathways [4]. The natural products with benzopyran skeleton have been demonstrated to have considerable bioactivities, including anti‐fungi [5], anti‐herbivore [6], and anti‐inflammation, which have been widely used as traditional medicine [7]. It is recently reported that xiamenmycin A can significantly attenuate hypertrophic scar formation and suppress local inflammation, which may serve as a potential medical candidate against excessive fibrotic disease [1–3]. Since fibrotic diseases, such as idiopathic pulmonary fibrosis, liver cirrhosis, systemic sclerosis, progressive kidney disease, and cardiovascular fibrosis, are some of serious threats to public health [8, 9]. Thus, identifying bioactive molecules that can reduce fibrosis and improving the production for pharmaceutical investigation have become an urgent topic.

It is well known that the production level of most secondary metabolites is always too low to satisfy the industrial production [10]. To fulfil the requirements of pharmaceutical investigations, a large‐scale culture and high production for secondary metabolites are now top of the priorities in the research. However, a bottleneck in bioengineering of secondary metabolites is lack of *in silico* model which can guide the enhancement of production. In this paper, we present a kinetic model for benzopyran production in *Streptomyces*.

Some *in silico* works on metabolic network modelling based on flux balance analysis (FBA) have been applied to the bacteria aiming for strain improvement with desired properties [10–12]. Although FBA can be a good method to obtain the optimal fluxes for biomass production, the production of secondary metabolites does not fall into the objective as it does not coincide with rapid growth of *Streptomyces.* We use a new kinetic metabolic modelling approach in the present paper [10, 13].

Kinetic modelling of metabolic network is a potential useful tool to simulate the biological processes *in vivo* and predict the flux in various circumstances. Most importantly, it tests the time‐dependent dynamics of metabolites against the steady solutions obtained in the modelling. Traditionally, kinetic modelling is hindered by the numerous unknown enzymatic parameters. In this paper, we have employed two methods to overcome the main obstacles. We first use generic enzymatic rate equation which standardises and reduces the parameters needed to construct a model. We then further imposed on the metabolic network a stability requirement by introducing regulations on the reaction parameters. The metabolic network is thus regulated in such a way that it adjusts to a set of parameters that guarantee its stability.

We are able to successfully obtain a set of parameters for genetically engineered *Streptomyces lividans* with glucose feed that are not only stable against metabolic fluctuations but show a reasonable amount of xiamenmycin. To further verify the *in silico* solution, we also simulated the strain growing on a combination of glucose and glycerol media. We found that xiamenmycin production rate is significantly higher in the combination, which is consistent with experiments.

The links between primary and secondary metabolisms in the biosynthesis can be explored. Phosphoenolpyruvate (PEP) is the key intermediate supporting xiamenmycin biosynthesis because the flux increases of PEP synthesis from 2‐phosphoglycerate (2‐PG) associates with the output flux of xiamenmycin. Adding glycerol as an extra carbon source is predicted to enhance xiamenmycin production. Calculations show glycerol may provide another carbon source for PEP without interfering TCA cycle. The framework established in this paper sets the foundation for more difficult tasks in the research. It can provide a blue print and ways to evaluate engineered *S. lividans* with xiamenmycin‐oriented genetic modification aiming to enhance xiamenmycin production.

## 2 Results and discussion

### 2.1 Construction of bioreaction network

*Streptomyces* is known for producing novel secondary metabolites and for highly effective processing of bioactive compounds. Xiamenmycin is one of the leading anti‐fibrotic compounds isolated from *S. xiamenensis* [14, 15]. The biosynthetic pathway has been elucidated encoded by a gene cluster named *Xim A–E* [16]. It was found that the biosynthetic pathway is closely related to central metabolism and starts with the formation of 4‐hyrobenzoic acid (4HB) by XimC [16]. The linkage of the geranyl side chain (GPP) to the benzene nucleus is catalysed by Xim B, then a possible epoxide intermediate is generated and cyclised by Xim D and Xim E. The forming of the subsequent amide bond connected the threonine (Thr) moiety with the benzopyran skeleton to finalise the biosynthesis of xiamenmycin, catalysed by Xim A. Three building metabolites, that is, 4HB, GPP, and Thr are produced by, respectively, shikimate pathway, methylerythritol phosphate (MEP) pathway, and threonine biosynthetic pathway. As a result, the key metabolites oxaloacetate (OAA) and PEP from citric acid (tricarboxylic acid (TCA)) cycle and glycolysis of glucose are the preferential sources to support the biosynthesis of xiamenmycin. The metabolic pathways necessary for energy balance and cell growth are included in our modelling. They are serine cycle, pentose phosphate pathway, gluconeogenesis, serine biosynthesis, and respiratory chain. The metabolic reaction network consists of 82 reactions and 86 metabolites. The metabolites and their corresponding abbreviation are listed in Table 2.

**Table 2:** List of metabolites in central metabolism and biosynthetic pathway for xiamenmycin

ID	Metabolite	Abbreviation	Biomass	
			Glucose	Glucose + Glycerol
1	nicotinamide adenine dinucleotide	NAD	0.8	0.8
2	nicotinamide adenine dinucleotide phosphate	NADP	0.2	0.2
3	adenosine triphosphate	ATP	−2.5	−2.5
4	serine	Ser	−0.03	−0.03
5	3‐phosphoglycerate	3‐PG	0	0
6	2‐phosphoglycerate	2‐PG	0	0
7	phosphoenolpyruvate	PEP	−0.05	−0.05
8	oxaloacetate	OAA	−0.2	−0.2
9	malate	Mal	0	0
10	acetyl‐CoA	Ac‐CoA	−0.2	−0.2
11	pyruvate	Pyr	−0.2	−0.2
12	coenzyme A	CoA	0.23	0.23
13	citrate	Cit	0	0
14	cis‐aconitate	Cis‐acon	0	0
15	isocitrate	Icit	0	0
16	alpha‐ketoglutarate	a‐KG	−0.07	−0.07
17	succinyl‐CoA	Succ‐CoA	−0.03	−0.03
18	succinate	Succ	−0.03	−0.03
19	fumarate	Fum	0	0
20	flavin adenine dinucleotide	FAD	0	0
21	glucose 6‐phosphate	G‐6‐P	0	0
22	fructose 6‐phosphate	F‐6‐P	−0.07	−0.07
23	fructose 1,6‐biphosphate	FBP	0	0
24	dihydroxyacetone phosphate	DHAP	0	0
25	glyceraldehyde 3‐phosphate	TP	−0.01	−0.01
26	1,3‐biphosphoglycerate	1,3‐BPG	0	0
27	6‐phosphogluconate	6‐PG	0	0
28	ribose 5‐phosphate	R‐5‐P	−0.05	−0.05
29	ribulose 5‐phosphate	Ru‐5‐P	0	0
30	xylulose 5‐phosphate	Xu‐5‐P	0	0
31	sedoheptolose‐7‐phosphate	S‐7‐P	0	0
32	erythrose‐4‐phosphate	E‐4‐P	−0.02	−0.02
33	glutamate	Glu	−0.07	−0.07
34	ubiquinone	Q	0	0
35	cytochrome‐c (oxidised)	Cyt‐Cox	0	0
36	nicotinamide adenine dinucleotide (reduced)	NADH	−0.8	−0.8
37	nicotinamide adenine dinucleotide phosphate (reduced)	NADPH	−0.2	−0.2
38	adenosine diphosphate	ADP	2.5	2.5
39	flavin adenine dinucleotide (reduced)	FADH2	0	0
40	ubiquinol	QH2	0	0
41	cytochrome‐c (reduced)	Cyt‐Cred	0	0
42	carbon dioxide	CO_2_	0	0
43	ammonium	NH_4_	0.06	0.1
44	phosphate	Pi	0.19996	0.2
45	membrane‐bound proton	H	0	0
46	D‐glycerol‐3‐phosphate	glycrol‐3P	0	0
47	glycerol	glycerol	0	0
48	3‐deoxy‐D‐arabino‐heptulosonate‐7‐phosphate	DAHP	0	0
49	3‐dehydroquinate	DHQ	0	0
50	3‐dehydroshikimate	DHS	0	0
51	shikimate	shikimate	0	0
52	shikimate 3‐phosphate	shikimate‐3P	0	0
53	5‐enolpyruvyl‐shikimate 3‐phosphate	EPSP	0	0
54	chorismate	chorismate	0	0
55	4‐Hydroxybenzoic acid	4HB	0	0
56	3‐octaprenyl‐4‐hydroxybenzoic acid	G‐4HB	0	0
57	xiamenmycin B	XiaB	0	0
58	xiamenmycin A	XiaA	−0.05	−0.07
59	geranyl pyrophosphate	GPP	0	0
60	L‐threonine	Thr	0	0
61	adenosine monophosphate	AMP	0	0
62	1‐deoxy‐D‐xylulose 5‐phosphate	DXP	0	0
63	2‐C‐methyl‐D‐erythritol 4‐phosphate	MEP	0	0
64	4‐(cytidine 5′‐diphospho)‐2‐C‐methyl‐D‐erythritiol	CDP‐ME	0	0
65	2‐phopho‐4‐(cytidine 5′‐diphospho)‐2‐C‐methyl‐D‐erythritol	CDP‐MEP	0	0
66	2‐C‐methyl‐D‐erythritol 2,4‐cyclodiphosphate	ME‐CPP	0	0
67	1‐Hydroxy‐2‐methyl‐2‐(E)‐butenyl 4‐diphosphate	HMBPP	0	0
68	isopentenyl diphosphate	IPP	0	0
69	dimethylallyl diphosphate	DMAPP	0	0
70	cytidine‐triphosphate	CTP	0	0
71	cytidine‐5′‐monophosphate	CMP	0	0
72	cytidine‐diphosphate	CDP	0	0
73	oxidised ferredoxin	ox‐FD	0	0
74	reduced ferredoxin	re‐FD	0	0
75	L‐aspartate	Asp	0	0
76	L‐aspartate 4‐phosphate	Asp‐4P	0	0
77	L‐aspartate‐semi‐aldehyde	Asp‐SA	0	0
78	L‐homoserine	HSER	0	0
79	O‐phospho‐L‐homoserine	PSER	0	0
80	glucose	Glc	0	0
81	acetate	Acetate	0	0
82	3‐phosphohydroxypyruvate	PHP	0	0
83	3‐Phosphoserine	3Pser	0	0
84	glutamine	Gln	0	0
85	guanosine triphosphate	GTP	0	0
86	guanosine diphosphate	GDP	0	0
1000	external network	external	−0.00057	−2.75×10^−14^

### 2.2 In silico simulation of xiamenmycin production

**Fig. 1:**
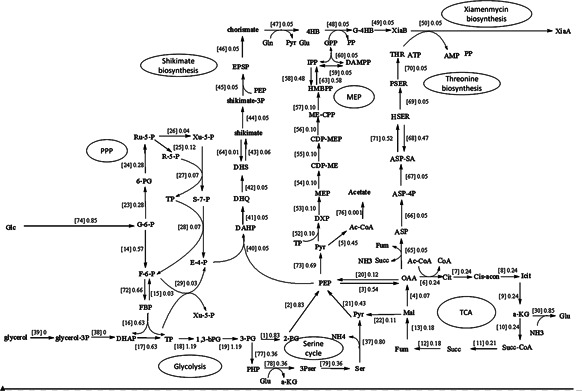
Main metabolic pathways and fluxes for production of xiamenmycin using glucose as carbon source. (A total of eight pathways are presented for xiamnemycin biosynthesis from glucose, containing 82 reactions and 86 metabolites. Prediction of fluxes is given for each reactions. Abbreviations of metabolites are listed in Table 2.)

Our purpose is to understand the bioprocess of secondary metabolites and provide guidance for optimising the production of xiamenmycin biosynthesis including the flux distribution and identification of the key reactions. From our kinetic simulation, the flux distributions feed on various carbon sources were predicted (Table 1) and the carbon efficiency was used as a criterion for evaluating the production. By setting the value of biomass as a fixed number, we simulated the metabolic behaviour under the same growth rate. The predicted steady‐state flux distribution using glucose as preferential carbon source was shown in Fig. 1 when growing on GYM (glucose 4 g/l, yeast extract 4 g/l, malt extract 10 g/l, and pH 7.2–7.4) medium.

**Table 1:** Kinetic parameters for the dynamical model

ID	Equation	Flux	
		Glucose	Glucose + glycerol
1	2‐PG ↔ 3‐PG	−0.82659	−0.91367
2	2‐PG ↔ PEP	0.82659	0.91367
3	PEP + CO_2_ ↔ OAA + Pi	0.54279	0.5468
4	OAA + NADH ↔ Mal + NAD	−0.07568	−0.0712
5	Pyr + CoA + NAD → Ac‐CoA + CO_2_ + NADH	0.44552	0.43047
6	Ac‐CoA + OAA → Cit + CoA	0.24442	0.22959
7	Cis‐acon ↔ Cit	−0.24442	−0.22959
8	Icit ↔ Cis‐acon	−0.24442	−0.22959
9	Icit + NADP → a‐KG + NADPH + CO_2_	0.24442	0.22959
10	a‐KG + 2 ox‐FD + CoA → Succ‐CoA + CO_2_ + 2 re‐FD	0.24442	0.22959
11	Succ + ATP + CoA ↔ Succ‐CoA + Pi + ADP	−0.21442	−0.19959
12	Fum + FADH2 ↔ Succ + FAD	−0.18442	−0.16959
13	Fum ↔ Mal	0.18442	0.16959
14	G‐6‐P ↔ F‐6‐P	0.57208	0.46316
15	FBP → F‐6‐P + Pi	0.027647	0.036677
16	FBP ↔ DHAP + TP	0.63255	0.48471
17	TP ↔ DHAP	−0.63274	−0.91497
18	TP + Pi + NAD ↔ 1,3‐BPG + NADH	1.1855	1.2505
19	3‐PG + ATP ↔ 1,3‐BPG + ADP	−1.1855	−1.2505
20	OAA + GTP → PEP + GDP + CO_2_	0.12405	0.11841
21	PEP + ADP ↔ Pyr + ATP	−0.4302	−0.44886
22	Mal + NAD → Pyr + CO_2_ + NADH	0.10875	0.098384
23	G‐6‐P + NADP → 6‐PG + NADPH	0.2807	0.23234
24	6‐PG + NADP → Ru‐5‐P + CO_2_ + NADPH	0.2807	0.23234
25	R‐5‐P ↔ Ru‐5‐P	−0.15023	−0.14078
26	Ru‐5‐P ↔ Xu‐5‐P	0.13046	0.091559
27	Xu‐5‐P + R‐5‐P ↔ S‐7‐P + TP	0.10023	0.090779
28	S‐7‐P + TP ↔ F‐6‐P + E‐4‐P	0.10023	0.090779
29	Xu‐5‐P + E‐4‐P ↔ F‐6‐P + TP	0.030232	0.000779
30	Glu + NADP ↔ NH_4_ + a‐KG + NADPH	−0.51131	−0.51318
31	NADH + Q → NAD + QH2 + 2H	1.3859	1.5046
32	QH2 + 2 Cyt‐Cox → Q + 2 Cyt‐Cred	1.5703	1.6742
33	FADH2 + Q → FAD + QH2	0.18442	0.16959
34	ATP ← ADP + 2 H + Pi	−2.3392	−2.5443
35	Cyt‐Cred → Cyt‐Cox + H	3.1406	3.3484
36	NADH + NADP + H ↔ NADPH + NAD	1.0896	1.1795
37	Ser → Pyr + NH_4_	0.32892	0.30679
38	glycrol‐3P + NAD → DHAP + NADH	0.000189	0.43026
39	glycerol + ATP → glycrol‐3P + ADP	0.000151	0.43026
40	PEP + E–4–P → DAHP + Pi	0.05	0.07
41	DAHP → DHQ + Pi	0.05	0.07
42	DHQ ↔ DHS	0.05	0.07
43	DHS + NADPH ↔ shikimate + NADP	0.057585	0.024537
44	ATP + shikimate ↔ ADP + shikimate‐3P	0.05	0.07
45	PEP + shikimate‐3P ↔ Pi + EPSP	0.05	0.07
46	EPSP → chorismate + Pi	0.05	0.07
47	chorismate + Gln ↔ 4HB + Pyr + Glu	0.05	0.07
48	GPP + 4HB ↔ G‐4HB + 2 Pi	0.05	0.07
49	G‐4HB + NADPH ↔ XiaB + NADP	0.05	0.07
50	XiaB + Thr + ATP ↔ XiaA + AMP + 2 Pi	0.05	0.07
51	ATP + AMP ↔ 2 ADP	−0.63914	−0.67503
52	TP + Pyr → DXP + CO_2_	0.1	0.14
53	DXP + NADPH ↔ MEP + NADP	0.1	0.14
54	MEP + CTP ↔ CDP‐ME + 2 Pi	0.1	0.14
55	CDP‐ME + ATP ↔ CDP‐MEP + ADP	0.1	0.14
56	CDP‐MEP ↔ ME‐CPP + CMP	0.1	0.14
57	ME‐CPP + 2 re‐FD ↔ HMBPP + 2 ox‐FD	0.1	0.14
58	HMBPP + NADH ↔ IPP + NAD	−0.47853	−0.41127
59	IPP ↔ DMAPP	0.05	0.07
60	DMAPP + IPP ↔ GPP + 2 Pi	0.05	0.07
61	CMP + ATP ↔ CDP + ADP	0.1	0.14
62	CDP + ATP ↔ CTP + ADP	0.1	0.14
63	HMBPP + NADPH ↔ IPP + NADP	0.57853	0.55127
64	shikimate + NAD ↔ DHS + NADH	0.007586	−0.04546
65	OAA + NH_4_ + Succ ↔ Asp + Fum	0.05	0.07
66	Asp + ATP ↔ Asp‐4P + ADP	0.05	0.07
67	Asp‐4P + NADPH ↔ Asp‐SA + NADP + Pi	0.05	0.07
68	Asp‐SA + NADH ↔ HSER + NAD	−0.47041	−0.41116
69	HSER + ATP ↔ PSER + ADP	0.05	0.07
70	PSER → Thr + Pi	0.05	0.07
71	Asp‐SA + NADPH ↔ HSER + NADP	0.52041	0.48116
72	F–6–P + ATP → FBP + ADP	0.66019	0.52139
73	Pyr + ATP ↔ PEP + AMP + Pi	−0.68804	−0.74415
74	ATP + Glc ↔ ADP + G–6–P	0.85278	0.69549
75	2 re‐FD + NAD + H ↔ 2 ox‐FD + NADH	0.14442	0.089588
76	Ac‐CoA + 2 Pi + AMP ↔ acetate + ATP + CoA	0.001093	0.000877
77	3‐PG + NAD ↔ PHP + NADH	0.35892	0.33679
78	PHP + Glu → a‐KG + 3Pser	0.35892	0.33679
79	3Pser → Ser + Pi	0.35892	0.33679
80	a‐KG + Gln + NADPH ↔ 2 Glu + NADP	−0.17238	−0.17639
81	Glu + NH_4_ ↔ Gln	−0.12238	−0.10639
82	ATP + GDP ↔ ADP + GTP	0.12405	0.11841
1001	ATP → ADP + Pi	2.5	2.5
1002	NADPH → NADP	0.2	0.2
1003	NADH → NAD	0.8	0.8
1004	external ← CO_2_	−1.0051	−0.93198
1005	external ← Ac‐CoA	−0.2	−0.2
1006	external ← Pyr	−0.2	−0.2
1007	external ← OAA	−0.2	−0.2
1008	external ← a‐KG	−0.07	−0.07
1009	external ← Glu	0.07	0.07
1010	external ← F‐6‐P	−0.07	−0.07
1011	external ← PEP	−0.05	−0.05
1012	external ← R‐5‐P	−0.05	−0.05
1013	external ← Succ‐CoA	−0.03	−0.03
1014	external ← Ser	−0.03	−0.03
1015	external ← E‐4‐P	−0.02	−0.02
1016	external ← TP	−0.01	−0.01
1017	external → Glc	0.85278	0.69549
1018	external ← XiaA	−0.05	−0.07
1019	external ← acetate	−0.00109	−0.00088
1020	external ← Succ	−0.03	−0.03
1021	external → glycerol	0	0.43026
1022	external → 4HB	0	0

**Fig. 2:**
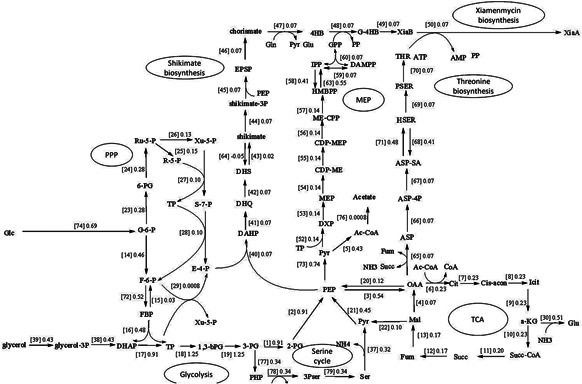
Main metabolic pathways for production of xiamenmycin using glucose and glycerol as mixture carbon source

**Fig. 3:**
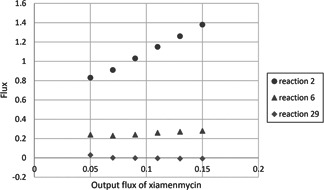
Changes of selected fluxes using glucose and glycerol as mixture carbon source

Glycerol is one of the carbon sources for *Streptomyces* metabolism which can support the fastest growth rate for *S. lividans* [17]. We investigated the glycerol plus media for improvement of xiamenmycin production *in silico* since *S. xiamenesis* also contained the *glpCABX* genes for glycerol utilisation. Thus, glycerol may serve as another carbon source when we try to improve the compositions of medium. We simulated the flux distribution when growing on mixture carbon sources (Fig. 2 and Table 1). Theoretically, the maximum efficiency of xiamenmycin production is 20% on GYM media (glucose), 27% on GYM (glucose) plus glycerol media as total. The efficiency is 28.8% on glucose in the combined media. While maintaining the same growth rate, adding glycerol as an extra carbon source is predicted to enhance xiamenmycin production by 40%. As shown in Fig. 3, PEP is the key intermediate supporting xiamenmycin biosynthesis because the flux increases of PEP synthesis from 2‐PG (reaction 2) associates with the output flux of xiamenmycin (reaction 50). Calculations show glycerol may provide another carbon source for PEP without interfering TCA cycle through glycerol degradation pathway to enter the glycolysis by seven steps, which is glycerol → glycerol‐3P → DHAP → TP → 1,3–bPG → 3‐PG → 2‐PG → PEP.

### 2.3 Discussion

In the metabolic engineering aspect, increase of productivity has been investigated experimentally and theoretically. There are still many problems need to be solved. One of the difficulties of experimental design is to find possible bottlenecks of metabolic network [18]. There are a lots of possible metabolic feedback loops that we can modify by genetic strategies for metabolic engineering, including increasing the precursor supply, overexpressing or increasing the efficiency of bottleneck enzyme, altering the regulation of gene expression, reducing flux toward unwanted by products or competing pathway, and reconstructing entire pathways in a heterologous host [18]. To predict the production and flux under various circumstances and provide a rational guidance for experiments, kinetic modelling of metabolic network is a straight forward solution for us.

However, previously due to the complexity of the biological systems and the diverse of enzymatic rate equation, it is impossible to manually adjust the parameters of kinetic model. Our kinetic model of large‐scale metabolic network based on a generic enzymatic rate equation [19]. In the generic form, kinetic parameters are reduced to a manageable level [20]. The generic rate equation is symmetrical in both directions of reversible reaction and formally exact under the quasi‐steady‐state condition. We used the carbon metabolism of *Methylobacterium extorquens* AM1 as a model study [13].

Even with reduced parameters, since there are complex regulations and non‐linear function, the steady solutions for the kinetic model found by trial and error is not possible for large‐scale network. Previously, there was no standard method for systematical adjustments. We demonstrated that a dynamical network may be stabilised with a simple forward form of regulation constructed on Lyapunov function derived from stochastic [21].

## 3 Materials and methods

### 3.1 Generic enzymatic rate equation

Though the large number of parameters in our kinetic model make it difficult to determine all the parameters experimentally, the full knowledge of mechanistic reaction rates is not always necessary in order to correctly characterise the behaviour of the organism. This is because physiologically metabolite concentrations are usually restricted to a rather narrow subspace of the whole range [13]. Moreover, enzymes catalyse most biological reactions. Therefore, we are able to construct the kinetic model based on generic enzymatic rate equation with a minimum set of parameters, see supplementary material I.

A chemical reaction can be written in the general form of (1)

(1) $$A_{1}+A_{2}+\cdots +A_{m}\overset{V_{F}}{\underset{V_{B}}{⇄}}P_{1}+P_{2}+\cdots +P_{n}$$

Each *A_i_* or *P_i_* can be the same substrate as the previous *A_i_* or they can be a different metabolite. In this way, the stoichiometry is specified. Implicit in the above is an enzyme which appears unbound on both sides of the reactions [13, 21, 22].

A generic enzymatic rate equation can be written in the general form of (2)

(2) $$\begin{array}{rl} & u\left(\left[A_{i}\right],\;\left[P_{j}\right]\right)=\frac{V_{F}\prod_{i=1}^{m}\left(\left[A_{i}\right]/K_{i}\right)-V_{B}\prod_{\;j=1}^{n}\left(\left[P_{j}\right]/K_{j}\right)}{\;f_{1}\left(V_{F},\;V_{B}\right)\prod_{i=1}^{m}\left(1+\left\{\left[A_{i}\right]/K_{i}\right\}\right)+\;f_{2}\left(V_{F},\;V_{B}\right)\prod_{\;j=1}^{n}\left(1+\left\{\left[P_{j}\right]/K_{j}\right\}\right)}\; \end{array}$$

The two functions of *f*_1_ and *f*_2_ have the following properties, see (3)–(5)

(3) $$\;f_{1}\left(V_{F},\;V_{B}\right)+\;f_{2}\left(V_{F},\;V_{B}\right)=1$$

(4) $$\;f_{1}\left(V_{F}=0,\;V_{B}\right)=0\;$$

(5) $$\;f_{1}\left(V_{F},\;V_{B}=0\right)=0\;$$

To summarise, we use generic rate equation for our calculations. This general form of enzymatic rate equation requires a minimum set of parameters, that is, the maximal forward and backward reaction velocities (*V*_F_ and *V*_B_), and the Michaelis–Menten‐like parameters (*K_i_*).

### 3.2 Metabolic optimisation by network Lyapunov function

We introduce a mathematic method to stabilise a dynamic network with a simple and straight forward form of regulation constructed based on Lyapunov function derived from stochastic dynamics, see supplementary material II.

Consider a metabolic network with *N* metabolites whose dynamics can be described by (6)

(6) $$\frac{dx}{dt}=Su-b=f(x,\;V)\;$$

with metabolite concentrations ***x*** and parameter set ***V***. ***S*** is the stoichiometric matrix and ***b*** is a vector containing inputs and outputs to the system as well as maintenance energy requirements, generally named as boundary [23]. We used the precursor requirements in *M. extorquens* AM1 adjusted to our growth rate of *Streptomyces* [23].

We found that the regulatory dynamics, as given by (7) and (8)

(7) $$W(x,\;V)\frac{dV}{dt}=-\nabla_{v}\psi (x,\;V)$$

(8) $$\psi (x,\;V)=-\nabla_{x}\phi (x,V)\cdot f(x,\;V)\;$$

stabilises the original network (6). Here ***W*** (***x***, ***V***) is a positive‐definite modulation matrix and *φ(x*, *V)* is a Lyapunov function for the metabolites dynamical network [21, 22]. The steady‐state solutions are then presented in this paper. In our previous publication,(7) was used to obtain suitable parameters *V*_f_ and *V*_b_ [21, 22]. This equation is assumed to govern a dynamical system whose Lyapunov function takes the form of psi = ***ψ***, in which ***S*** scales inversely with diffusion matrix ***D***. In our calculation, we assumed a simple diagonal form of ***D*** and ***S***, by assuming smaller metabolite compounds having bigger ***D***, inversely linked to the number of carbon chain. We used *V*_f_ = 0.4, *V*_b_ = 0.4 as initial values. In this range, different initial values result in slightly output fluxes of slightly different carbon efficiencies. In (7), we set ***W*** = **1**. Using these input values, (7) is solved by MATLAB software package ode23t for *t* = 0:100,000 s. The output files are (Metabolites_f, Reactions_f) for calculation represented in Table 1. More information was provided in supplementary material II.

*V*_f_ and *V*_b_ for reactions were optimised by calculations using (7) and (8), as explained more in supplementary material II. Glucose and glutamate were used as the nutrition in GYM media. Glycerol was added as another carbon source afterwards as a comparison. Biomass is defined as flux exchanges between the metabolites considered in the model and the rest not included in our model, which can be estimated according to the growth rate. The values of biomasses for *Streptomyces* have been increased by ten times, compared with *M. extorquens* AM1, due to different growth rate. Xiamenmycin A was used as our target output compound, whose biomass was set to be −0.05 accordingly.

## 4 Conclusions

The primary focus of metabolic engineering is on enhancement of productions. On the basis of an optimised kinetic metabolic modelling, we demonstrate *in silico* that a significant improvement on the production of xiamenmycin can be obtained when adding glycerol feed as an additional carbon source. The flux distributions of genetically engineered *S. lividans* were calculated. In addition, our modelling offers a practical approach to deal with unavailable kinetic parameters for metabolic network modelling.
